# Integrated Core Proteomics, Subtractive Proteomics, and Immunoinformatics Investigation to Unveil a Potential Multi-Epitope Vaccine against Schistosomiasis

**DOI:** 10.3390/vaccines9060658

**Published:** 2021-06-16

**Authors:** Abdur Rehman, Sajjad Ahmad, Farah Shahid, Aqel Albutti, Ameen S. S. Alwashmi, Mohammad Abdullah Aljasir, Naif Alhumeed, Muhammad Qasim, Usman Ali Ashfaq, Muhammad Tahir ul Qamar

**Affiliations:** 1 Department of Bioinformatics and Biotechnology, Government College University, Faisalabad 38000, Pakistan; abdurrehman93@gcuf.edu.pk (A.R.); farahshahid24@gcuf.edu.pk (F.S.); qasimawan@gcuf.edu.pk (M.Q.); ashfaqua@gcuf.edu.pk (U.A.A.); 2 Department of Health and Biological Sciences, Abasyn University, Peshawar 25000, Pakistan; sajjad.ahmad@abasyn.edu.pk; 3 Department of Medical Biotechnology, College of Applied Medical Sciences, Qassim University, Buraydah 51452, Saudi Arabia; as.albutti@qu.edu.sa; 4 Department of Medical Laboratories, College of Applied Medical Sciences, Qassim University, Buraydah 51452, Saudi Arabia; Aswshmy@qu.edu.sa (A.S.S.A.); Mjasr@qu.edu.sa (M.A.A.); 5 Ministry of Education, Riyadh 11153, Saudi Arabia; Naifalhumeed@hotmail.com; 6 College of Life Science and Technology, Guangxi University, Nanning 530004, China

**Keywords:** schistosomiasis, *Schistosoma japonicum*, *Schistosoma mansoni*, *Schistosoma haematobium*, multi-epitope vaccine, MD simulation

## Abstract

Schistosomiasis is a parasitic infection that causes considerable morbidity and mortality in the world. Infections of parasitic blood flukes, known as schistosomes, cause the disease. No vaccine is available yet and thus there is a need to design an effective vaccine against schistosomiasis. *Schistosoma japonicum*, *Schistosoma mansoni*, and *Schistosoma haematobium* are the main pathogenic species that infect humans. In this research, core proteomics was combined with a subtractive proteomics pipeline to identify suitable antigenic proteins for the construction of a multi-epitope vaccine (MEV) against human-infecting *Schistosoma* species. The pipeline revealed two antigenic proteins—calcium binding and mycosubtilin synthase subunit C—as promising vaccine targets. T and B cell epitopes from the targeted proteins were predicted using multiple bioinformatics and immunoinformatics databases. Seven cytotoxic T cell lymphocytes (CTL), three helper T cell lymphocytes (HTL), and four linear B cell lymphocytes (LBL) epitopes were fused with a suitable adjuvant and linkers to design a 217 amino-acid-long MEV. The vaccine was coupled with a TLR-4 agonist (RS-09; Sequence: APPHALS) adjuvant to enhance the immune responses. The designed MEV was stable, highly antigenic, and non-allergenic to human use. Molecular docking, molecular dynamics (MD) simulations, and molecular mechanics/generalized Born surface area (MMGBSA) analysis were performed to study the binding affinity and molecular interactions of the MEV with human immune receptors (TLR2 and TLR4) and MHC molecules (MHC I and MHC II). The MEV expression capability was tested in an *Escherichia coli* (strain-K12) plasmid vector pET-28a(+). Findings of these computer assays proved the MEV as highly promising in establishing protective immunity against the pathogens; nevertheless, additional validation by in vivo and in vitro experiments is required to discuss its real immune-protective efficacy.

## 1. Introduction

Schistosomes are parasitic flatworms that cause an infectious disease called schistosomiasis [1]. Schistosomiasis is one of the most common and neglected tropical parasite disorders in developing countries. Schistosomiasis is the second leading cause of death due to parasitic diseases, accounting for around 200,000 deaths per year according to the World Health Organization (WHO) [2]. Infection occurs when the freely floating larvae, cercariae, released by different snail intermediate hosts, penetrate the skin of a human host after contact with contaminated fresh water [3].

*Schistosoma japonicum*, *Schistosoma mansoni*, and *Schistosoma haematobium* are the most common *Schistosoma* species capable of infecting humans [4]. *S. mansoni* infections cause intestinal/hepatic schistosomiasis in Brazil, Sub-Saharan Africa, Venezuela, Puerto Rico, the Republic of Suriname, and the Caribbean islands [5]. *S. haematobium* causes urogenital schistosomiasis in the Middle East and Sub-Saharan Africa, specifically, Yemen, Egypt, and Sudan, while *S. japonicum*, a zoonotic trematode, causes intestinal/hepatic disease (Oriental Asiatic or schistosomiasis) in the Philippines, Indonesia, and China [5].

Currently, the majority of schistosomiasis control is achieved by prescribing praziquantel (PZQ) to infected people. Even though PZQ is highly effective against adult schistosome parasites, using it as a monotherapy raises the likelihood of drug failure as drug-resistant parasites could emerge. Furthermore, since PZQ does not prevent re-infection, significant efforts and infrastructure to control schistosomiasis are required [6]. Other preventative steps, such as hygiene (WaSH) systems, water sanitation, and intermediate snail host control, also has had little impact [7]. Despite these concerted and massive efforts, active schistosomiasis is now reported in formerly schistosomiasis-free geographic areas [8]. Given the aforementioned circumstances, it is clear that to make significant progress toward long-term schistosomiasis control, integrated safety measures, with an effective vaccine playing a key role, are required [9]. The development of the vaccine against schistosomiasis would be a very effective plan to combat it.

Biologically prepared vaccines are often an option because prophylactic methods are better than therapeutic methods [10]. Vaccines based on conventional whole organisms composed of killed organisms or live attenuated ones contain large, unwanted, antigenic determinants, which lead to unspecified or uncertain immune responses [11]. Similarly, subunit vaccines consisting of one protein usually cause allergic reactions and high undesirable antigenic loads [12,13]. The vaccines based on epitopes are instead safe, produced easily, immune-specific, and chemically stable [14]. However, peptide vaccines, with poor T cell population coverage, are weakly immunogenic [13]. Multi-epitope peptide vaccines (MEVs), which link antigenic peptides that activate both T and B cell immunity, are the most effective way to overcome these flaws [15]. Immunoinformatics approaches are a viable option for developing and designing highly immunogenic MEVs. Immunoinformatics has recently been used to design MEVs against viruses [16,17,18,19,20,21], parasites [22,23], and bacteria [24,25,26,27,28].

The study presented here used a subtractive proteomics method to prioritize subunit vaccine candidates from the core proteome of *Schistosoma* species, followed by immunoinformatics analysis to forecast the T and B cell epitopes. The prioritized B cell and T cell epitopes were used in docking and simulation studies that determine the affinity of the MEV construct for the TLR2 and TLR4 receptor, as well as to look for conformational changes in the receptor and MEV that affect construct binding. We assume that the designed vaccine will be useful for vaccine professionals to test its immune-protective potential and effectiveness in controlling *Schistosoma* infections in animal models.

## 2. Materials and Methods

### 2.1. Identification of the Schistosoma Core Proteome

The reference proteomes of three *Schistosoma* species (*S. haematobium* (SchHae_2.0), *S. japonicum* (ASM636876v1), and *S. mansoni* (ASM23792v2)) were retrieved from the genome database of NCBI and subjected to core genome analysis using an in-house, Perl-written program language script. Fast clustering of the proteomes was achieved by setting a sequence identity cut-off of 50%. An output file containing the core protein sequences shared by all the species was considered for vaccine designing as these sequences are conserved across strains and species and categorized as broad-spectrum vaccine candidates [29].

### 2.2. Subtractive Proteomics Approach

A subtractive proteomic approach was used for analyzing the core proteome to recognize suitable vaccine candidates. Subtractive proteomics is a computational method for identifying potential vaccine and drug targets by excluding proteins that are not useful for vaccine and drug designing [30]. The first step in subtractive proteomics was to find duplicated sequences in the core proteome that shared an 80% sequence identity using the CD-HIT algorithm [31]. Following that, the non-redundant proteins were compared to the human host to eliminate homologous proteins and prevent functional blockage of similar host proteins. BLASTp against a reference human proteome with predetermined parameters was used to screen non-homologous proteins from a pool of non-redundant proteins [32]. The CELLO server was combined with the SVM method to predict the subcellular localization of non-host/homologous proteins with a prediction accuracy of 89% [33].

Virulent proteins play a key role in the development of vaccines due to their host invasion and pathogenesis nature. *Schistosoma* virulent proteins were identified by using ViroBLAST [34]. Furthermore, the antigenicity of the virulent proteins was analyzed using the Vaxijen server. Through ACC (auto cross-covariance) transformation, this server maintains a prediction accuracy of 70–89% [35]. An antigenic protein was defined as one with an antigenic score greater than 0.5. TMHMM was used with cut-off < 1 to predict transmembrane helices [36]. Proteins with fewer transmembrane helices are easier to express and clone [21]. Top antigenic proteins having 1 or 0 transmembrane helices were chosen for vaccine development. Moreover, the AllerTOP server evaluated the allergenicity of proteins. AllerTOP is a robust and powerful complementary approach based on the k-nearest neighbors (kNN) method for classifying non-allergens and allergens with 88.7% accuracy [37].

### 2.3. Prediction of Epitopes

#### 2.3.1. Prediction of CTL Epitopes

A significant breakthrough in rational vaccine design is the development of cytotoxic T-lymphocyte (CTL) epitopes. Most importantly, it decreases the time and expense of predicting epitopes compared to wet laboratory experiments [38]. CTL epitopes were predicted by using the MHC I binding tool present in the Immune Epitope Database (IEDB) [39]. The lesser percentile rank shows greater affinity, so the percentile rank was considered as 2. Immunogenic, non-toxic, non-allergen, and antigenic CTL epitopes were considered for vaccine construction. The immunogenicity tool present in IEDB was used to find the immunogenic nature of the epitopes [39]. The Toxin Pred and Vaxijen servers were used to check for toxicity and antigenicity [40,41]. The Toxin Pred server is based on the various properties of peptides and employs a quantitative matrix and machine learning technique. Allergenicity was checked using AllergenFP, which ensures a prediction accuracy up to 88.9% [42].

#### 2.3.2. Prediction of HTL Epitopes

Helper T lymphocyte (HTL) cells influence CTL cells to kill target parasitized cells, macrophages to ingest infectious agents, and B cells to excrete antibodies, making them the most effective cells in adaptive immunity [43]. Therefore, it is essential to include helper T cell epitopes to create a healthier immune reaction. Cytokines were generated in HTL cells, such as interleukin-4 (IL-4) and IL-10, and interferon-gamma (IFN-), which cause immune cells such as macrophages and cytotoxic T cells to become activated [44]. Hence, HTL epitopes that induce cytokine production are critical for vaccine creation. The MHC-II binding tool of IEDB was utilized to forecast HTL epitopes (15-mer) of the target proteins [39]. The IFN-epitope server was used with motif scan and SVM approaches to produce overlapping HTL sequence peptides against the query protein [45]. The IL-4Pred server enables to plan, detect, and map peptides that really can induce IL-4 with a threshold value of 0.2, which is important for subunit vaccines [46]. IL-10Pred was also used to predict the inducing properties at the threshold value of −0.3 [47].

#### 2.3.3. Prediction of LBL Epitopes

The surface receptor of B cell identifies B cell epitopes and produces antigen-related immunoglobulins. The formulation of a B cell epitopic vaccine would therefore play an important role in adaptive immunity [48]. The online server ABCPred, based on a neural networking approach, was used to identify the linear B cell epitopes (LBL) with an accuracy of 75% [49]. The AllergenFP v1.0, ToxinPred, and VaxiJen v2.0 servers were utilized to check the allergenicity, toxicity, and antigenicity of the anticipated B cell epitopes [35,40,42].

### 2.4. World Population Coverage

Different HLA alleles and their expression are distributed in different ethnic groups at various frequencies. The worldwide HLA-alleles distribution is therefore essential for successful multi-epitope vaccine development [50]. The main goal of these population coverage studies was to see if the candidates chosen were appropriate for large groups of people [51]. To determine the population coverage of the screened epitopes and their HLA alleles, the IEDB population coverage server was used [52].

### 2.5. MEV Construction

The potential CTL, HTL, and LBL epitopes were combined to form a fusion peptide using the AAY, GPGPG, and KK peptide linkers, respectively. Since the peptides used to make vaccines are generally not very immunogenic when used alone, adjuvants are mandatory to improve the immune reaction. Hence, the TLR-4 agonist (RS-09; Sequence: APPHALS) adjuvant was linked to the CTL epitope via the EAAAK linker. A linker must be used to join two epitopes for each epitope to work efficiently.

### 2.6. Structural Analysis of the Vaccine Construct

The constructed vaccine was initially tested using BLASTp for its similarity with the human proteome [53]. Next, the Protparam web server was used to evaluate the vaccine construct’s physiochemical characteristics [37]. Molecular weight, atomic composition, amino-acid composition, extinction coefficient, theoretical pI, and approximate half-life were the parameters determined by the ProtParam server [54]. In addition, the IEDB Immunogenicity server was utilized to calculate immunogenicity and the Vaxijen server was used to evaluate vaccine construct antigenicity (antigen or non-antigen) [39,41]. One more important step was to calculate the allergenicity (allergen or non-allergen) of the vaccine construct using the AllerTOP server [55]. Protein secondary structure is very significant because it is a key indicator of protein folding, so the SOPMA Tool evaluates the secondary structure of the vaccine. The secondary structure prediction by SOPMA shows an accuracy of 69.5% by showing a three-state structure (a-helix, B-sheet, and coil) description. [56]. The last step of the vaccine construct evaluation includes the SOLpro Tool for checking the vaccine solubility, which has a 74% accuracy with a 10-fold cross-validation [57].

### 2.7. 3D Structure Prediction and Validation

A 3D protein model can be created using computer algorithms from the amino acid sequence. The 3Dpro tool of the SCRATCH suite was used for the three-dimensional (3D) modeling of the MEV [58]. This server predicts the three-dimensional structures of the protein based on several-threading alignments and iterative prototype fragment assembly simulations. After the prediction of the vaccine model, it was refined using the GalaxyRefine webserver [59]. The GalaxyRefine webserver reconfigures the side chain and then uses molecular dynamics to carry out the structural reassembling and then the overall structural inspection. It is especially effective in improving the quality of the local structures, as demonstrated by the CASP refinement category. The Ramachandran plot was used to evaluate the energetically permissible and forbidden phi (ϕ) and psi (ψ) dihedral angles of the amino acid residues [60]. Besides, ProSA-web deals with specific needs in the validation of the protein structures derived from NMR spectroscopy, X-ray, and theoretical calculations [61]. ERRAT was also used to evaluate our predicted tertiary structure and assign a value of the quality factor based on the non-bonded atomic interactions of the various atomic types within the protein [62].

### 2.8. Prediction of the B Cell Epitopes of the Vaccine

The IEDB v2.22 Ellipro tool and ABCPred online server were used to predict the MEV’s conformational and linear B cell epitopes [49,63]. On the ABCPred server, the MEV amino acid sequence was given as input, with the length set to 14 and the threshold set to 0.5. The 3D structure of the MEV was also given as input in the Ellipro tool by keeping the default parameters.

### 2.9. In Silico Cloning and Codon Adaptation

A codon adaptation strategy can improve the foreign gene expression in the host. The use of codons varies from organism to organism, and this change in the use of codons results in low foreign gene expression. The codon adaptation of the vaccine sequence was performed through the JCAT tool and adapted to codon usage as per the *E.coli* K12 strain usage [64]. The adapted nucleotide sequence was cloned into an *E.coli* vector of pET28a (+) utilizing Snap Gene v5.0.8 software [65].

### 2.10. Immune Simulation

The C-IMMSIM server assesses the immune response and immunogenicity of the MEV. The C-IMMSIM server predicts the peptide interactions with the immune system employing position-specific scoring matrices (PSSM). The C-ImmSim server is routinely employed in immunoinformatics studies and gives reliable results with regard to the vaccination strategy employed [66,67,68]. The simulation was conducted in 1000 steps, with two doses given over a four-week interval.

### 2.11. Protein–Protein Docking

The vaccine interacts with host immune cells to provide an effective immune response. The HADDOCK v2.2 server was used to conduct the protein–protein docking to analyze the MEV binding capability with human Toll-like receptors (TLR-2 and TLR-4) and MHC molecules (MHC I and MHC II) [69]. HADDOCK is a (experimental) knowledge-based docking method in the form of information on the interface zone between the molecular components and related orientations. In contrast to many other docking programs, HADDOCK permits a conformational modification of molecules not only of the side chains but also of the backbone during the complex formation. Furthermore, HADDOCK supports the docking of multi-model NMR structures and other Protein Data Bank (PDB) structures [70]. Crystal structures of TLR4 (ID: 4G8A) [71], TLR2 (ID: 2Z7X) [72], MHC I (ID: 1I1Y) [73], and MHC II (ID: 1KG0) [74] were retrieved from the PDB [75]. Interactions were observed by evaluating docked complexes in the PDBsum database, and the docked complexes were imaged using PyMOL [76,77]. PDBsum is a web-based database that provides a large pictorial summary of the key data on each PDB macromolecular structure. It includes PROMOTIF structural studies, annotated plots of each protein chain’s secondary structure, images of the structure, PROCHECK results, and schematic diagrams of the protein–DNA and protein–ligand interactions [78].

### 2.12. MD Simulations

Simulations were used to understand the molecular dynamics of the docked complexes. The AMBER software and its various modules were used for this purpose [79]. Initially, the TLeap module was used for the generation of topological files and initial coordinates. The system was solvated utilizing the force field ff14SB in a TIP3P water box with 8.0 dimensions [80]. To recover adverse conflicts, the complex’s energy was minimized using the conjugate gradient for 1000 steps and the steepest descent. Using the Langevin dynamics algorithm, the device was then heated for 10 ps to preserve temperature stability. The pressure was balanced in accordance with protocol. Finally, the complex was subjected to an efficient simulation of 100 ns. The simulation box’s canonical ensemble inferred periodic boundary conditions. The Berendsen Coupling Integration Algorithm was employed to keep the temperature stable [81]. The Process TRAJectory (PTRAJ) module was used to analyze the results. Xmgrace was used to calculate and display three properties [82]. These are the radius of gyration (RoG), root mean square deviation (RMSD), and root mean square fluctuation (RMSF). Alpha carbon (Cα) co-ordinates are commonly considered to be depictive of an amino acid’s position in 3D space. The RMSD method compares the protein carbon atoms in relative positions by determining the average distance between them over a certain period of time [83]. RMSF calculates the protein’s backbone atoms (N, C, and C) and analyzes the structural changes. This reflects the root average square distance in certain dynamics between an atom and its average geometric position. RoG is used to evaluate the 3D packaging and density of the docked complexes [84].

### 2.13. MMGBSA Binding Energy Analysis

MMGBSA was used to estimate the binding free energies of the dominating simulated complexes. The ante-MMPBSA.py module generated and evaluated the initial prompt files for the MEBV, TLR2, TLR4, MHCI, MHCII, and complexes. The variation in the free energies of the complex, receptor, and the vaccine was used to calculate the free energy:

ΔGbind = (ΔGcomplex) − (ΔGreceptor + ΔGvaccine)(1)

The gas-phase energy influences are exchanged with polar and non-polar salvation free energy modules during this process [85]. For each terminus, MMGBSA calculates Gibb’s free energy, which is a term used to describe the amount of energy that is denoted by G, as follows:

ΔG = Egas + ΔGsolv − TS(2)

where temperature is denoted by T, and is multiplied by entropy S, which is estimated by normal mode analysis. The MM energy from the force field is often used as "Egas" at the gas stage energy. Van der Waals collaboration, internal, and electrostatic energy are all included in this category.

## 3. Results

### 3.1. Core Proteome Analysis

The core proteins are now appreciated in vaccine design as they are present in all or the majority of the targeted pathogen strains and the use of these proteins in vaccine design ensure immune protection against broader pathogenic species. For vaccine design against Schistosoma, all three prominent pathogenic species (*Schistosoma mansoni*, *Schistosoma japonicum*, and *Schistosoma haematobium*) were considered. The reference proteome of *Schistosoma japonicum* has a total size of 369.9 Mb encoding 16,936 proteins with GC contents of 33.8%. Similarly, Schistosoma mansoni and Schistosoma haematobium encodes for 3462 and 8934, respectively. The total protein count of these species is 29,332; which reduced to 16,557 after core proteome analysis.

### 3.2. Identification of Vaccine Candidates

The CD-hit study of the core proteome identified 11,430 proteins as non-redundant. The redundant proteins are the product of evolutionary duplication and are a copy of the core protein. Since these proteins are not conserved in the genome, they are not good candidates for vaccine development [86]. When screening vaccine proteins, homologous proteins must be removed because they cause host proteins cross-reactivity, resulting in auto-immune responses [87]. Homology analysis identified 2094 non-homologous proteins in the host, while 9336 homologous proteins were excluded. During comparative subcellular localization, CELLO identified 79 cytoplasmic, 612 plasma membrane, 184 extracellular, 102 mitochondrial, 1 cytoskeletal, and 1116 nuclear proteins. Since cytoplasmic proteins do not interact with the extracellular environment of the pathogen, these are not considered to be adequate vaccine design targets and therefore were discarded. The remaining proteins were subjected to virulent protein analysis. Virulent protein immunization and/or their combinations enhance the host’s protection when exposed to a microbial challenge [14]. The virulent protein mediates severe signals in host cells and thus can serve as valuable vaccine candidates in comparison with non-virulent proteins. Four proteins were shortlisted as virulent proteins [88]. The antigenicity of these four proteins was then analyzed and only two proteins were found to be antigenic. Antigenicity is defined as the ability to elicit an immune response in response to an antigen; thus, selecting proteins with higher antigenicity is a requirement for peptide-based vaccine design [89]. Besides, no transmembrane helices were found in these two proteins. Furthermore, these proteins were found to be non-allergenic, making them prime candidates for vaccine development. Immunoinformatics analysis of both these proteins was done. Details of both proteins are listed in Table 1.

### 3.3. Epitopes Prediction

Vaccine candidates were used to forecast CTL, HTL, and LBL cell epitopes. The top 7 non-toxic, antigenic, non-allergenic, and immunogenic CTL epitopes were screened for vaccine designing, as shown in Table 2, from a total of 66 CTL epitopes forecasted (Table S1).

Similarly, a total of 10 HTL epitopes were forecasted (Table S2) and top 3 antigenic, IFN-positive, IL-10 inducer, and IL-4 inducer HTL epitopes were screened for the vaccine construct, as shown in Table 3.

B cell epitopes are protein antigenic regions that can trigger the formation of antibodies. A total 23 LBL epitopes were predicted (Table S3). However, only the top 4 antigenic, non-allergic, and non-toxic LBL epitopes with a probability score above 0.5 were considered for the MEV design, as shown in Table 4.

### 3.4. World Population Coverage

**Figure 1:**
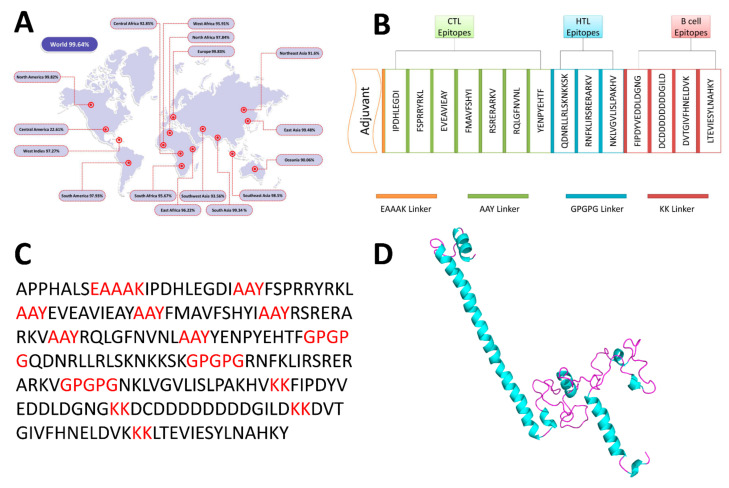
Different vaccine design analyses performed in this study. (**A**) Worldwide population coverage of MEV epitopes. (**B**) Schematic representation of the final vaccine construct components. (**C**) Primary sequence of the MEV, where a red color represents linkers and black color represents adjuvant and epitopes. (**D**) 3D model of the MEV.

The distribution of MHC alleles varies across the geographical regions and ethnic groups. Hence, population coverage must be considered while developing an effective vaccine [50]. Population coverage of the screened T cell epitopes was checked (Figure 1A). the global population of selected epitopes is estimated to be 99.64%. The highest population coverage was found in Europe (99.83%), followed by North America (99.82%), East Asia (99.48%), and South Africa (99.34%). Central America has shown the lowest population coverage (22.61%).

### 3.5. Construction of the MEV

The MEV is a fusion peptide that was designed by using AAY, KK, and GPGPG linkers to combine the 10 T cell (7 CTL and 3 HTL epitopes) and 4 LBL epitopes (Figure 1B). The TLR-4 agonist (RS-09; Sequence: APPHALS) was added to activate an antigen-specific immune response with an EAAAK linker in the N-terminal of the vaccine. The final vaccine contained a total of 217 amino acid residues (Figure 1C).

### 3.6. Physiochemical and Structural Analysis of the Vaccine Construct

After the development of a vaccine, different physiochemical and immunogenic properties were analyzed. Initially, the vaccine homology was checked, and it was found to be non-homologous. Further, the antigenicity of the construct was calculated at a threshold value of 0.5 and found to be an antigen with a score of 0.7222. In the next step, allergenicity and toxicity were also checked, and the vaccine was observed to be non-toxic and non-allergenic.

The physiochemical properties were evaluated by the Protparam server. The MEV was estimated to have a theoretical pI of 8.90 and a molecular weight of 24.3 kDa. The MEV consists of 32 negatively charged amino acid residues (Asp + Glu) and 36 positively charged amino acid residues (Arg + Lys). A large number of positively charged amino acids and a pI greater than 7 showed that the MEV was alkaline. A 29.76 Instability Index (II) classified the MEV as stable. The MEV’s thermostability was indicated by an Aliphatic Index of 78.29. The MEV’s GRAVY score was −0.655; a negative sign indicates that it is hydrophilic. Finally, the SOLpro tool was used to estimate MEV solubility and the results indicated that MEV was soluble (probability: 0.952236). Transmembrane helices were checked by the TMHMM server and one transmembrane helix was found in the MEV at the position of 40–59 amino acid residues. Each of these characteristics suggests that the designed MEV has a high probability of being accepted as a vaccine candidate.

SOPMA was used to examine the secondary structure of the MEV. The SOPMA predictions are based on the MEV amino acid sequence. SOPMA results indicate that 68 (31.34%) amino acids form coils, 101 (46.54%) amino acids form an α-helix, and 30 (13.82%) amino acids forms β-strands.

The 3Dpro server was used for the tertiary structure prediction of the MEV. GalaxyRefine was used to further refine the predicted 3D structure of the MEV. The 3D structure of the MEV is shown in Figure 1D. GalaxyRefine returned five refined structures. Model 2 was chosen for further validation among the pool because it had a significantly higher Rama favored region (95.9) and overall suitable MolProbity (1.730), RMSD (0.483), and GDT-HA (0.9278) scores.

A Ramachandran plot of the vaccine subunit revealed that 94.6% of the amino acid residues were present in the favored region, 4.3% in the additional allowed region, and 0.5% in the disallowed region, which corresponds to GalaxyRefine’s prediction. A Z-score of −2.85 was predicted by the ProsA web server. For high-quality models, ERRAT generates an overall quality factor of >50, and our model’s quality factor was 76.84.

### 3.7. Prediction of B Cell Epitopes of the MEV

B cells also produce antibodies, which give humoral immunity and release cytokines. Therefore, B cell epitopes should be present in the MEV’s domain. Seven conformal B cell epitopes (Table S4) and sixteen linear B cell epitopes (Table S5) were forecasted.

### 3.8. In Silico Cloning

**Figure 2:**
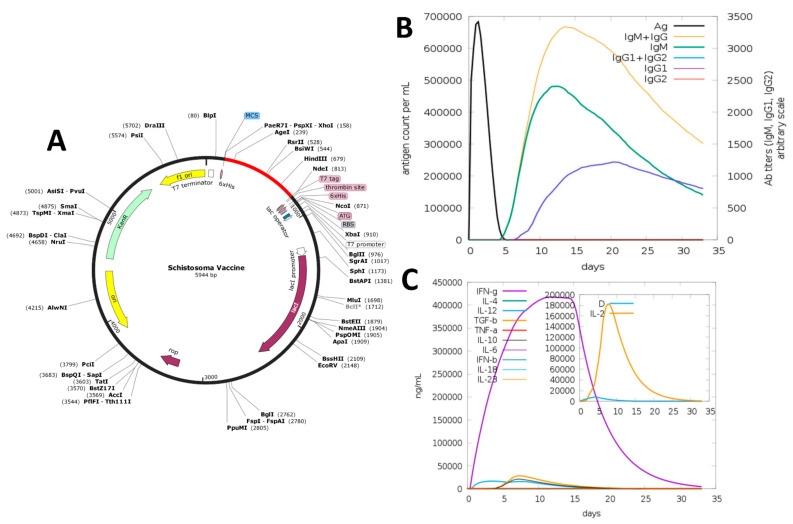
In silico cloning and immune simulation studies of the MEV: (**A**) cloned sequence of the MEV (colored as red) in the pET28a (+) expression vector; (**B**) immunoglobulins response per mL to the presence of the MEV antigen; (**C**) interferon and interleukins concentration in ng/mL generated in response to MEV.

To express the vaccine peptide in an *E. coli* system, in silico cloning of the MEV was performed in the pET28a (+) plasmid. The cloned sequence and the plasmid were cleaved with the restriction enzymes (Ndel and Xhol) to produce flanking sites. Before that, the construct sequence’s codon usage was adapted as per the *E.coli* K12 strain’s usage. JCAT revealed that the improved sequence exhibits a GC content of 50.0% and a CIA value of 1. To aid in the purification process, the 6-histidine residues were labeled on both sides of the target sequence. The clone was 5944 bp in size (Figure 2A).

### 3.9. Immune Simulation

The C-IMMSIM web server was used to test the immunogenic profile of the designed vaccine. All primary, secondary, and tertiary immune responses contributed significantly to the vaccine immunity (Figure 2B). In particular, high titers of IgG + IgM antibodies were observed, followed by IgM and IgG1. Furthermore, in response to vaccine administration, different B cell isotypes were developed, which resulted in memory cell development. Furthermore, the vaccine candidate induces high IFN-γ and IL-2 levels (Figure 2C).

### 3.10. Protein–Protein Docking

A vaccine must have a high binding affinity to the immunological receptors of the host, such as MHC molecules and Toll-like receptors, to elicit proper immune responses. Protein–protein docking of the MEV protein was performed with MHC molecules (MHC I and MHC II) and human receptors (TLR4 and TLR2) using the HADDOCK v.2.4 server. Binding scores of the MEV-TLR2, MEV-TLR4, MEV-MHC I, and MEV-MHC II complexes were 170.5 ± 17.0 kcal/mol, 84.7 ± 38.6 kcal/mol, 73.8 ± 18.3 kcal/mol, and 86.3 ± 33.0 kcal/mol, respectively, as shown in the Table 5. According to the docking statistics, the MEV has strong binding interactions with TLR2, TLR4, MHC I, and MHC II.

**Figure 3:**
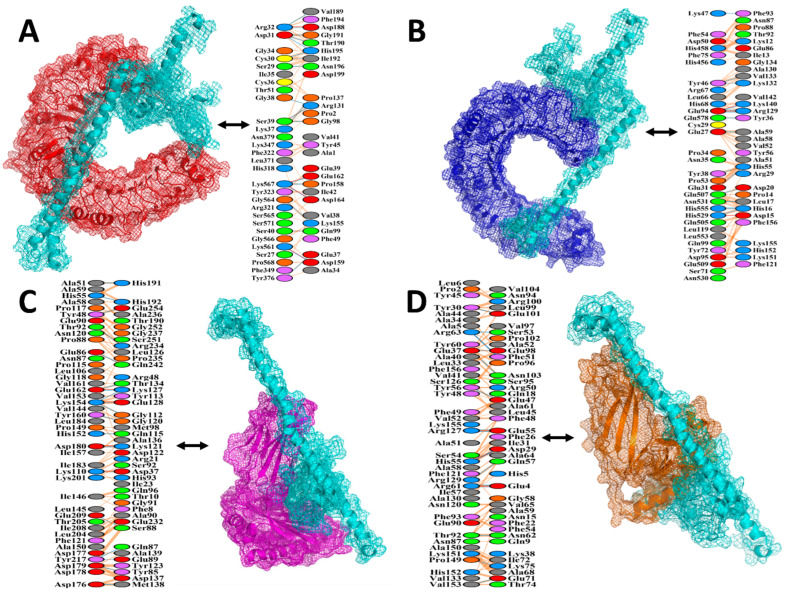
MEV–immune receptors binding conformation and interaction analysis. Intermolecular binding mode and residue level chemical interactions of (**A**) MEV-TLR2; (**B**) MEV-TLR4; (**C**) MEV-MHC I; (**D**) MEV-MHC II. The MEV is shown in cyan mesh, whereas TLR2, TLR4, MHC I, and MHC II are presented via mesh firebrick, blue, magenta, and sienna, respectively.

The MEV docked conformation and atomic-level hydrogen bonding with different immune receptors are illustrated in Figure 3. The PDBsum server was used to gain a better understanding of the interactions between the MEV and receptor molecules and to draw up an interaction map between the docked complexes. It resulted in a schematic representation of non-bonded and H-bond interactions between the docked complexes. The MEV had 19 hydrogen bonds with TLR2 in the range of 3.07 Å, 15 hydrogen bonds with TLR4 in the range of 3.08 Å, 19 hydrogen bonds with the MHC I receptor in the range of 3.13 Å, and 17 hydrogen bonds with the MHC II receptor in the range of 3.27 Å.

### 3.11. MD Simulation

**Figure 4:**
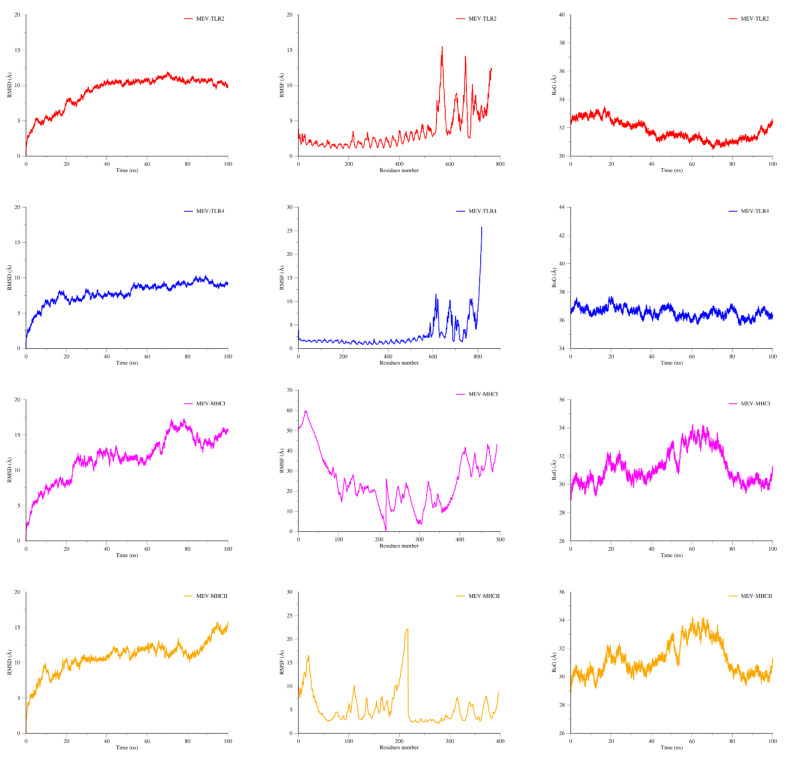
Molecular dynamics simulation-based statistical analysis to evaluate the intermolecular stability and dynamics of the complexes.

The statistical parameters based on the 100 ns MD simulation RMSF and RMSD were determined for the docked complexes (MEV-TLR4, MEV-TLR2, MEV-MHCI, and MEV-MHCII) to confirm the docked and structural stability of the constructed MEV (Figure 4). Simulation trajectories were used in both studies, and charts for the backbone carbon Alpha atoms were also created for both RMSF and RMSD. The RMSD backbone steadily increases in the complexes over time, and visualizing frames at 10 ns intervals revealed that the varying plot corresponds to slight structural changes induced, due to flexible loop regions, by the MEV. The binding of TLR2, TLR4, MHCI, and MHCII, as well as the overall stability of the complexes, were unaffected by these changes. The mean RMSD values for the MEV-TLR2, MEV-TLR4, MEV-MHC I, and MEV-MHC II complexes were 9.9 Å, 7.9 Å, 12.3 Å, and 12.4 Å, respectively. The RMSF analysis showed that the complex flexibility is due to the MEV’s loop flexibility, which is higher for the MEV compared to the receptor molecules. An RMSF of TLR2 showed stability up to 2.5 Å. RMSF of TLR4 showed good conformational stability up to 2.3 Å. MHC I showed minor deviation at some points while MHC II showed stability at the value of 3 Å; after minor deviation, it remained stable at 2.5 Å. This indicated that the MEV in complex with TLR receptors are more stable than MHC molecules. The compactness of the complexes was then studied through the 100 ns simulation by measuring the RoG. The TLR2 receptor showed stability up to 32.5 Å over the time period of 35 ns; after that, it showed a minor deviation and remained stable up to 100 ns with the MEV receptor. The MHC-I and MHC-II plots of the RG indicate that they showed stability between 30 Å with minor deviations of 1 Å over the time period of 100 ns. RG conformational stability of the TLR4 receptor with MEV showed stability over 36.9 Å. All three these statistical analyses validated that the MEV is enjoying more stable dynamics with the TLR receptors than with the MHC molecules; therefore, it can be predicted that the MEV is more likely to bind to the TLRs.

### 3.12. MMGBSA Binding Energy Analysis

The MMGBSA method was used to calculate the binding free energies of the docked complexes and is represented in Table 6. The total binding energies of the MEV-TLR2, MEV-TLR4, MEV-MHCI, and MEV-MHCII complexes were −86.24 kcal mol^−1^, −91.47 kcal mol^−1^, −149.25 kcal mol^−1^, and −124.13 kcal mol^−1^, respectively.

Based on the calculated values, it could be concluded that van der Waals energy and electrostatic energy seem to be more beneficial in complex formation than the non-polar portion of the solvation energy’s minor contribution. Overall, polar solvation energy tends to be less favorable to net energy.

## 4. Discussion

Schistosomiasis, the second most common tropical disease following malaria, has been a threat to the residents in regions it is endemic. In light of the possibility of resistance to the widely used drug (praziquantel) for schistosomiasis treatment, the need for a long-term vaccination strategy has been justified [90]. Vaccines are important for inducing immune protection and high immune responses against a variety of infectious diseases. Traditional approaches for vaccine production are less capable than computational methods for a variety of reasons, including inaccuracy, safety, stability, high cost, hypersensitivity, and specificity [91]. The subtractive proteomics approach in combination with immuno-informatics recently became more attractive for the designing of an effective low-cost vaccine. A substantial amount of genomic information available allows us to identify the pathogen proteins that are suitable for vaccine designing [91,92].

Prioritizing potential vaccine candidates requires the presence of a virulent factor capable of causing severe host infection [88,93]. Subcellular localization, no transmembrane helices, allergenicity, and antigenicity are also reported parameters that may help to screen potential vaccine candidates [14]. In the current study, subtractive proteomics in combination with reverse vaccinology and molecular docking was applied to identify and evaluate epitope-based antigenic peptide proteins in the core proteome of *Schistosoma* species. Subtractive proteomics was used to thoroughly investigate the core proteome to identify non-homologous, non-redundant, non-allergenic, virulent, and antigenic vaccine candidates. Two proteins—calcium binding and mycosubtilin synthase subunit C—were identified as promising vaccine candidates. Further, epitopes were predicted from these proteins. It has been revealed that nucleus localized proteins can enter endogenous pathways and are targets of the class I processing pathway [94,95,96]. Another way of using these vaccine candidates is in the form of a DNA vaccine comprising integrated antigens. The development of such multivalent DNA vaccines is a novel approach in parasite vaccinology where multiple antigens are combined into a plasmid or administered as plasmid mixture. This type of vaccination also allows protection against a variety of parasite strains as well as against different life cycle stages of parasites. The efficacy of these vaccines against parasites can be enhanced by achieving prime-boost immunizations, use of genetic adjuvants, and codon optimization [97].

Both B and T cell epitopes are required to concatenate and induce both humoral and cellular immunity when a prolonged, significant immune response is desired. Another benefit of using both types of epitopes is that antigens can sometimes elude memory B cells, making identification difficult. In such cases, the contingency plan is that the T cells neutralize and recognize the missed epitopes or antigens so that no pathogen remains are found. For T cells, CD8+ (MHC-I) and CD4+ (MHC-II) epitopes were forecasted to have a greater number of potential epitopes capable of generating an increased immune response [98]. Since linear epitopes are more stable than conformational epitopes, only linear epitopes were predicted for B cell epitope prediction [99]. Hence, both T and B cell epitopes from vaccine candidates were forecasted and rigorously analyzed. The selected epitopes covered 99.64% of the population of the world.

KK, AAY, and GPGPG linkers between the epitope sequences were added to make a more coherent and stable vaccine construct [100,101]. The use of EAAAK as a linker enhances the bioactivity of the vaccine and was added to the N-terminus of the vaccine, based on previous studies [102]. The TLR-4 agonist (RS-09; Sequence: APPHALS) was included as an adjuvant. The involvement of RS09 allows CTL epitopes to be co-stimulated, resulting in a more robust immune activation. The use of synthetic adjuvants (RS09) is a safer approach that is regarded as an advancement over traditional vaccination methods [103]. RS09 serves as an excellent adjuvant and it has been used in many previous studies [104,105,106]. The final MEV is 217 amino-acids long, which is consistent with previous studies [107,108]. The results, therefore, suggested that the efficacy, expression, and stability of our vaccine would not be an issue. The physiochemical analysis of the MEV revealed that it is stable and hydrophilic.

Tertiary and secondary structures provide information about a protein’s function, interactions with other proteins/ligands, and dynamics [109,110]. Secondary structure analysis indicates that the MEV has a good amount of beta-sheets (30%), alpha-helixes (27%), and coils (41%), which is consistent with its capability to bind to immune cells and antigenicity [111]. A Ramachandran plot is an easy and simple way to analyze the tertiary structure quality. The presence of >90% residues in the Ramachandran plot (favored region) indicates high model quality, and our model has 94.6 residues in the favored region, indicating the high quality of our vaccine model [112]. Furthermore, the ERRAT quality factor and Z-score of our MEV were 76.84 and −2.85, respectively. A quality factor greater than 50% indicates that the model is of high quality [113]. The quality factor and Z-score confirm our model’s overall high quality.

The MEV construct was used to predict the B cell epitopes to see if it had enough epitopes for antibodies to detect and latch onto [114]. Furthermore, elevated vaccine construct expression in the *E. coli* system is necessary for serological assays to detect the vaccine candidate’s immunoreactivity [115]. For efficient expression in *E.coli*, the sequence was optimized. The optimized sequence has a CAI (Codon Adaptation Index) value of 1.00 and a GC content of 50%, indicating further purification and excellent expression of the vaccine [116,117].

Since MEV includes B and T cell epitopes, it should trigger cellular and humoral immune responses. Among other cytokines, IFN-β production was the highest. Important IL-10 and IL-2 activities were also observed. Extracellular protection was also provided by the antibodies. A large number of active immunoglobulins, including IgG and IgM, and their isotypes, have also been found, which can contribute to isotype switching. Furthermore, the negligible Simpson Index (D) indicates a varied, plausible immune response, as the MEV includes various B and T cell epitopes.

For MEV to be transported into the body successfully, it must have a high binding affinity for the immune receptor [118]. The MEV’s strong binding ability with MHC molecules (MHC-1 and MHC-2) is required to elicit the immune system and develop a vaccine and immunotherapy directed against infectious microorganisms. In response to the administrated specific epitopic antigens, these interactions initiate an innate immune response and then elicit an adaptive immune response [119,120]. MD simulation and molecular docking verified the strong interactions of MEV with TLR2, TLR4, MHC I, and MHC II; the MMGBSA study demonstrated that this stable binding required very little energy. A significant number of H-bonds in docking, as well as minor fluctuations during MD simulations were observed. According to these findings, the MEV can bind to immune receptors effectively.

Although some multi-epitope vaccines against *Schistosoma* have been reported [104,121,122], the MEV developed in this work exhibits outstanding properties because it uses the core proteome of three *Schistosoma* species that primarily affects humans. As the constructed vaccine contains an adjuvant, B cell, and T cell epitopes (CTL, HTL), it can promote innate and adjuvant immunological reactions in the host body, making it an excellent and suitable candidate for *Schistosoma* vaccine production. The only drawback of the current research is that further laboratory trials are required to demonstrate the safety and efficacy of the designed vaccine since it is based on an integral computation pipeline.

## 5. Conclusions

The availability of a complete parasitic proteome facilitates several computational methods. The results presented here exhibit the gradual prioritization of the core proteome using various reverse vaccinology and comparative proteomics approaches, thus lessening the undesirable number of *Schistosoma* proteins. This method is effective for enriching potential targets and identifying those that are essential for normal cell functions as well as those that are virulent in the host cell. This approach enables us to identify immunogenic and antigenic vaccine targets that are important for pathogenesis. Antigenic, non-allergenic, and non-toxic T cell and B cell epitopes were fused with several linkers and adjuvants to boost immune reactions that further improve the effectiveness of the designed vaccine. Nevertheless, the methods applied in the study focused on the use of stringent parameters to make the predictions as accurate as possible; however, the pipeline used herein suffers from several limitations. For example, predictions about the MEV epitopes to MHC alleles and B cells need extensive validation, although their reliability has been proved widely. An appropriate order of the epitopes in the MEV is required to be investigated experimentally in the context of immunogenicity. Moreover, to confirm its worth against *Schistosoma* infections, the designed vaccine needs to be validated in animal models.

## Figures and Tables

**Table 1:** Details of the *Schistosoma* vaccine candidates.

Accession No	Protein Name	Subcellular Localization	Antigenicity	TMHMM Helices
TNN18811.1	Calcium binding	Nuclear	0.6922	0
TNN05631.1	Mycosubtilin synthase subunit C	Nuclear	0.5741	0

**Table 2:** Screened CTL epitopes for MEV construction.

CTL Epitopes	Protein	Position	Corresponding Alleles	Antigenicity	Immunogenicity
IPDHLEGDI	Calcium binding	32–40	HLA-C*08:02	1.3173	0.16476
			HLA-B*51:01		
			HLA-B*53:01		
FSPRRYRKL	Calcium binding	152–160	HLA-B*14:02	1.0381	0.01308
			HLA-C*07:02		
			HLA-E*01:01		
			HLA-B*08:01		
			HLA-C*06:02		
EVEAVIEAY	Calcium binding	285–293	HLA-A*26:01	0.7113	0.34471
			HLA-A*25:01		
			HLA-A*01:01		
			HLA-B*35:01		
FMAVFSHYI	Mycosubtilin synthase subunit C	373–381	HLA-A*02:01	1.1695	0.03765
			HLA-A*02:06		
			HLA-A*68:02		
			HLA-A*29:02		
			HLA-B*39:01		
			HLA-A*32:01		
			HLA-A*24:02		
			HLA-B*46:01		
RSRERARKV	Mycosubtilin synthase subunit C	463–471	HLA-C*15:02	1.9596	0.12246
			HLA-C*06:02		
			HLA-A*30:01		
			HLA-C*07:01		
RQLGFNVNL	Mycosubtilin synthase subunit C	514–522	HLA-B*48:01	1.5715	0.16947
			HLA-A*32:01		
			HLA-B*40:01		
			HLA-A*02:06		
			HLA-B*27:05		
			HLA-B*39:01		
			HLA-B*40:02		
YENPYEHTF	Mycosubtilin synthase subunit C	669–677	HLA-B*18:01	1.1243	0.12737
			HLA-B*44:02		
			HLA-B*40:01		
			HLA-C*07:02		
			HLA-B*40:02		
			HLA-B*40:02		
			HLA-B*38:01		
			HLA-A*23:01		

**Table 3:** Screened HTL epitopes for MEV construction.

HTL Epitopes	Protein	Position	Alleles	Antigenicity	IL4/IL10	IFN
QDNRLLRLSKNKKSK	Calcium binding	333–347	HLA-DRB1*04:26	1.1906	Inducer	Positive
			HLA-DRB1*11:01			
			HLA-DRB1*04:21			
			HLA-DRB5*01:01			
			HLA-DRB1*04:02			
RNFKLIRSRERARKV	Mycosubtilin synthase subunit C	457–471	HLA-DRB5*01:01	1.0836	Inducer	Positive
			HLA-DRB5*01:05			
			HLA-DRB1*08:04			
			HLA-DRB1*11:01			
			HLA-DRB1*08:13			
			HLA-DRB1*08:06			
NKLVGVLISLPAKHV	Mycosubtilin synthase subunit C	680–694	HLA-DRB1*01:01	0.8180	Inducer	Positive
			HLA-DRB1*04:04			
			HLA-DRB1*09:01			
			HLA-DRB5*01:01			
			HLA-DRB1*15:01			
			HLA-DRB1*12:01			

**Table 4:** Screened LBL epitopes for MEV construction.

Peptide	Protein	Position	Score	Antigenicity	Immunogenicity
FIPDYVEDDLDGNG	Calcium binding	358–371	0.89	1.6532	0.23092
DCDDDDDDDDGILD	Calcium binding	537–550	0.61	1.7767	0.29686
DVTGIVFHNELDVK	Mycosubtilin synthase subunit C	96–109	0.84	1.1856	0.4856
LTEVIESYLNAHKY	Mycosubtilin synthase subunit C	644–657	0.66	0.8240	0.05747

**Table 5:** Docking parameters of the MEV with immune receptors and MHC molecules.

Docking Statistics	MEV-TLR2	MEV-TLR4	MEV-MHC I	MEV-MHC II
Cluster size	6	7	18	22
HADDOCK score	170.5 ± 17.0	84.7 ± 38.6	73.8 ± 18.3	86.3 ± 33.0
RMSD from the overall Lowest Energy Structure	13.2 ± 0.1	1.0 ± 0.8	3.8 ± 0.2	0.9 ± 0.6
Restraints violation energy	3336.6 ± 113.8	3416.1 ± 411.3	3071.8 ± 126.1	3073.3 ± 188.1
Electrostatic energy	−388.8 ± 78.9	−484.1 ± 54.5	−527.2 ± 80.2	−325.9 ± 41.6
Van der Waals energy	−67.4 ± 7.5	−97.6 ± 3.9	−96.0 ± 13.9	−104.6 ± 11.5
Buried surface area	2914.2 ± 152.2	3658.5 ± 91.0	4338.3 ± 271.5	4135.9 ± 163.5
De-solvation energy	−18.0 ± 5.2	−62.5 ± 9.6	−32.0 ± 2.5	−51.2 ± 5.2
Z-score	−1.7	−1.8	−1.0	−1.7

**Table 6:** Binding energies of the MEV to the human receptors and MHC molecules.

Energies	MEV-TLR2 (kcal/mol)	MEV-TLR4 (kcal/mol)	MEV-MHC I (kcal/mol)	MEV-MHC II (kcal/mol)
vdW	−69.19	−59.77	−91.74	−85.01
Ele	−29.07	−45.00	−59.12	−44.11
Polar solvation	46.68	51.2	33.22	44.7
Non polar solvation	−34.66	−37.9	−31.61	−39.71
∆Gas	−98.26	−104.77	−150.86	−129.12
∆Solvation	12.02	13.3	1.61	4.99
∆total	−86.24	−91.47	−149.25	−124.13

## Data Availability

The data presented in this study are available within the article.

## Supplementary Materials

The following are available online at https://www.mdpi.com/article/10.3390/vaccines9060658/s1, Table S1: CTL Epitopes predicted by the IEDB Consensus Method. Table S2: HTL Epitopes predicted by the IEDB Consensus Method. Table S3: LBL Epitopes predicted by ABCPred. Table S4: Conformational B cell epitopes in the vaccine predicted by ElliPro Server. Table S5: Linear B cell epitopes predicted in the vaccine by the ABCPred server.
