# Supplementary material for: Integrated Core Proteomics, Subtractive Proteomics, and Immunoinformatics Investigation to Unveil a Potential Multi-Epitope Vaccine against Schistosomiasis
Source: Vaccines (Basel). 2021 Jun 16;9(6):658. doi: 10.3390/vaccines9060658 (PMC8235758; doi:10.3390/vaccines9060658)
Supplement: Supplementary file 1 [file vaccines-09-00658-s001.zip › vaccines-1252803-supplementary.pdf]

Table S1 CTL Epitopes predicted by IEDB Consensus Method

Protein				
Calcium Binding				
Epitopes	Position	Alleles	Antigenicity	Immunogenicity
EVEAVIEAY	285-293	HLA-A*26:01<br>HLA-A*25:01<br>HLA-A*01:01<br>HLA-B*35:01	0.7113	0.34471
FSPRRYRKL	152-160	HLA-B*14:02<br>HLA-C*07:02<br>HLA-E*01:01<br>HLA-B*08:01<br>HLA-C*06:02	1.0381	0.01308
YLDEDYLHS	387-395	HLA-C*05:01	0.8030	0.13561
DSDHDGIPN	267-275	HLA-C*08:02<br>HLA-C*05:01	0.5176	0.19909
LEDEDHDGI	318-326	HLA-B*40:01<br>HLA-C*05:01	0.9741	0.19852
IPDHLEGDI	32-40	HLA-C*08:02<br>HLA-B*51:01<br>HLA-B*53:01	1.3173	0.16476
KQDNRLLRL	332-340	HLA-B*48:01<br>HLA-A*02:06	0.8049	0.06153
VEVEAVIEA	284-292	HLA-B*40:02<br>HLA-B*18:01	0.7580	0.36193
DSDGDGVLD	498-506	HLA-C*05:01<br>HLA-C*08:02	1.7229	0.17921
DKDTNNNGM	294-302	HLA-C*05:01	1.2454	0.04821
ESDDDDDDD	685-693	HLA-C*05:01	2.2082	0.10368
DSDGDGILD	181-189	HLA-C*08:02<br>HLA-C*05:01	1.4040	0.20064
DTDDDETNN	700-708	HLA-C*05:01	1.3095	0.17435
EGDINHNGI	37-45	HLA-C*05:01	2.0346	0.17961
EDDLDGNGI	364-372	HLA-C*05:01<br>HLA-C*08:02	2.2002	0.06388
QFSPRRYRK	151-159	HLA-A*31:01	1.2006	0.06138

		HLA-C*04:01		
YDDDDDNDG	474-482	HLA-C*05:01	1.7125	0.0795
FYDDDDDND	473-481	HLA-C*08:02	1.1230	0.08694
KQDRRHHKI	604-612	HLA-A*32:01	1.5880	0.04143
AKDTDGDGI	514-522	HLA-C*08:02<br>HLA-C*05:01	1.3628	0.13828
DDDIDHAHY	736-744	HLA-C*05:01	0.7508	0.24509
DRRHHKIAN	606-614	HLA-B*14:02	1.0397	0.01303
DIHEDENKN	550-558	HLA-A*25:01	1.4391	0.09564
Mycosubtilin synthase subunit C				
RQLGFNVNL	514-522	HLA-B*48:01<br>HLA-A*32:01<br>HLA-B*40:01<br>HLA-A*02:06<br>HLA-B*27:05<br>HLA-B*39:01<br>HLA-B*40:02	1.5715	0.16947
FIDPHTNHL	262-270	HLA-C*08:02<br>HLA-A*02:06<br>HLA-B*39:01<br>HLA-C*06:02<br>HLA-C*05:01<br>HLA-C*07:02<br>HLA-C*07:02	0.5218	0.07752
RSRERARKV	463-471	HLA-C*15:02<br>HLA-C*06:02<br>HLA-A*30:01<br>HLA-C*07:01	1.9596	0.12246
IVLTARYYY	661-669	HLA-A*29:02<br>HLA-A*30:02<br>HLA-A*11:01<br>HLA-B*57:01	1.1714	0.117
YENPYEHTF	669-677	HLA-B*18:01<br>HLA-B*44:02<br>HLA-B*40:01<br>HLA-C*07:02<br>HLA-B*40:02<br>HLA-B*40:02<br>HLA-B*38:01	1.1243	0.12737

		HLA-A*23:01		
RYYYENPYE	666-674	HLA-C*14:02<br>HLA-A*30:01	0.5529	0.07497
FMAVFSHYI	373-381	HLA-A*02:01<br>HLA-A*02:06<br>HLA-A*68:02<br>HLA-A*29:02<br>HLA-B*39:01<br>HLA-A*32:01<br>HLA-A*24:02<br>HLA-B*46:01	1.1695	0.03765
DPHTNHLYI	264-272	HLA-B*51:01<br>HLA-B*53:01	0.7871	0.06219
VNGIRFHAS	282-290	HLA-B*08:01<br>HLA-B*08:01	0.5288	0.35568
YYENPYEHT	668-676	HLA-C*14:02<br>HLA-C*07:02	1.0707	0.11511
KLVGVLISL	681-689	HLA-A*02:06<br>HLA-A*02:01<br>HLA-B*48:01<br>HLA-A*32:01	1.1142	0.09292
VLVNAFIEK	622-630	HLA-A*11:01<br>HLA-A*03:01	0.9063	0.32601
SRERARKVL	464-472	HLA-B*14:02<br>HLA-C*07:02<br>HLA-B*39:01<br>HLA-C*06:02	1.2369	0.01352
HMNENRNTM	346-354	HLA-C*07:02<br>HLA-B*39:01<br>HLA-B*35:01	0.9323	0.15829
LLSTQIVAI	727-735	HLA-A*32:01<br>HLA-A*02:01	0.5295	0.05554
TVLATEQLR	506-514	HLA-A*68:01	0.9125	0.06358
NVNLDVFTK	519-527	HLA-A*68:01<br>HLA-A*11:01	1.4741	0.16868
KYFQITHIT	19-27	HLA-C*14:02<br>HLA-A*23:01	0.6024	0.19264
YICGRTNEL	271-279	HLA-A*25:01<br>HLA-C*06:02<br>HLA-B*08:01<br>HLA-B*39:01	0.5753	0.15658
IIDVLVNAF	619-627	HLA-A*32:01<br>HLA-B*46:01<br>HLA-A*01:01	0.9993	0.0942
PKDDEDFYL	490-498	HLA-B*38:01	0.5501	0.24454

		HLA-E*01:03		
QQLINLTKY	12-20	HLA-A*30:02	0.5046	0.02034
EPNDNHEKI	357-365	HLA-B*51:01<br>HLA-B*53:01	0.9947	0.00287
KNDDFMAVF	369-377	HLA-C*05:01<br>HLA-B*58:02<br>HLA-C*04:01	0.6749	0.03536
GFNVNLDVF	517-525	HLA-A*23:01<br>HLA-C*04:01<br>HLA-B*46:01	1.7873	0.06554
IQGTAICIV	137-145	HLA-A*02:06	0.5705	0.2457
MEDEWYLSS	583-591	HLA-B*18:01	1.1215	0.21415
QTVHGRDLK	330-338	HLA-A*68:01<br>HLA-A*11:01	1.3044	0.13991
VLSVGTPIQ	130-138	HLA-E*01:01	1.2292	0.12578
AKQGYAAVV	774-782	HLA-B*39:01	0.5217	0.08687
KVNGIRFHA	281-289	HLA-A*30:01	1.3148	0.32802
DLKLVCFYV	336-344	HLA-E*01:03	0.7469	0.00493
QYLSPTFIN	383-391	HLA-E*01:03<br>HLA-A*23:01	0.9109	0.03223
TGKIGSILK	528-536	HLA-A*30:01	1.0987	0.04703
LTEVIESYL	644-652	HLA-A*01:01	0.7447	0.15611
NPYEHTFVK	671-679	HLA-B*53:01<br>HLA-A*68:01	1.4480	0.29051
TNHLYICGR	267-275	HLA-A*68:01	1.6066	0.09532
CSREELLVN	311-319	HLA-C*15:02	1.0726	0.21937
CGRTNELIK	273-281	HLA-A*30:01	0.8446	0.21221
LIRSRERAR	461-469	HLA-A*31:01<br>HLA-B*14:02	1.3840	0.06152
YEIFIEQWN	599-607	HLA-B*40:01	1.3698	0.41651
IDPHTNHLY	263-271	HLA-A*29:02	0.8416	0.08148

LYICGRTNE	270-278	HLA-C*14:02	1.5607	0.09965

Table S2 HTL Epitopes predicted by IEDB Consensus Method

Protein						
Calcium binding						
HTL epitopes	Position	alleles	Antigenicity	IFN-Y	IL-4	IL-10
KQDNRLLRLSKNKKS	332-346	HLA-DRB1*04:26<br>HLA-DRB1*04:21<br>HLA-DRB1*11:01<br>HLA-DRB1*04:02	0.9238	Positive	IL4-inducer	IL10-inducer
QDNRLLRLSKNKKSK	333-347	HLA-DRB1*04:26<br>HLA-DRB1*11:01<br>HLA-DRB1*04:21<br>HLA-DRB5*01:01<br>HLA-DRB1*04:02	1.1906	Positive	IL4-inducer	IL10-inducer
Mycosubtilin synthase subunit C						
KCSRNFKLIRSRERA	454-468	HLA-DRB5*01:01<br>HLA-DRB5*01:05	0.4332	Positive	IL4-inducer	IL10-inducer
RNFKLIRSRERARKV	457-471	HLA-DRB5*01:01<br>HLA-DRB5*01:05<br>HLA-DRB1*08:04<br>HLA-DRB1*11:01<br>HLA-DRB1*08:13<br>HLA-DRB1*08:06	1.0836	Positive	IL4-inducer	IL10-inducer
SRNFKLIRSRERARK	456-470	HLA-DRB5*01:01<br>HLA-DRB5*01:05<br>HLA-DRB1*08:04<br>HLA-DRB1*11:01<br>HLA-DRB1*08:13<br>HLA-DRB1*08:06	1.0114	Positive	IL4-inducer	IL10-inducer
GKCSRNFKLIRSRER	453-467	HLA-DRB5*01:01<br>HLA-DRB5*01:05	0.7614	Positive	IL4-inducer	IL10-inducer
FKLIRSRERARKVLA	459-473	HLA-DRB5*01:01<br>HLA-DRB5*01:05<br>HLA-DRB1*08:04<br>HLA-DRB1*08:13<br>HLA-DRB1*08:06	0.8807	Positive	IL4-inducer	IL10-inducer
NKLVGVLISLPAKHV	680-694	HLA-DRB1*01:01<br>HLA-DRB1*04:04<br>HLA-DRB1*09:01	0.8180	Positive	IL4-inducer	IL10-inducer

		HLA-DRB5*01:01<br>HLA-DRB1*15:01<br>HLA-DRB1*12:01				
VGVLISLPAKHVPSR	683-697	HLA-DRB1*01:01<br>HLA-DRB1*15:01<br>HLA-DRB1*09:01<br>HLA-DRB1*11:01<br>HLA-DRB1*12:01	0.6887	Positive	IL4-inducer	IL10-inducer
EHTFVKNKLVGVLIS	674-688	HLA-DRB1*08:06<br>HLA-DRB1*13:02	1.4159	Positive	IL4-inducer	IL10-inducer

Table S3 LBL Epitopes predicted by ABCPred

Peptide	Protein	Score	Antigenicity	Immunogenicity
FIPDYVEDDLDGNG	calcium binding	0.89	1.6532	0.23092
DDQDDDDDNNGIKD	calcium binding	0.86	1.2587	0.07674
KDSDNDGIIDFYDD	calcium binding	0.85	0.6044	0.41776
DSDGDGIPDYLNND	calcium binding	0.85	0.8382	0.21302
DGIIDFYDDDDDND	calcium binding	0.83	0.5183	0.40974
GDGVPDYLDEDANS	calcium binding	0.83	0.5520	0.21816
SDGDGIPDYQEDSD	calcium binding	0.82	0.8082	0.10663
DGDGIPDYLDDDDD	calcium binding	0.81	0.5581	0.24146
LEGDINHNGIIDYL	calcium binding	0.80	1.3728	0.48626
VPDHLEEDSDGDGI	calcium binding	0.79	1.1378	0.17647
DDDNDDIPDHLDSD	calcium binding	0.78	0.8840	0.12741
NDGIPDDQDDDDDN	calcium binding	0.68	1.6097	0.16028
DDDDDGILDIHEDE	calcium binding	0.66	0.8358	0.48203
DSDGDGILDFLEDS	calcium binding	0.66	0.7632	0.43732
DHLETDDTDGDGII	calcium binding	0.65	0.7522	0.39779
DCDDDDDDDDGILD	calcium binding	0.61	1.7767	0.29686
GDSVPEDKVADRNH	calcium binding	0.61	0.8812	0.02522
DFLEDSDGDGIPDY	calcium binding	0.58	0.5002	0.19573
DVTGIVFHNELDVK	Mycosubtilin synthase subunit C	0.84	1.1856	0.4856
IDVLVNAFIEKDRL	Mycosubtilin synthase subunit C	0.82	0.6455	0.2613
FMPGDLGFIDPHTN	Mycosubtilin synthase subunit C	0.73	0.6452	0.37904
EQLRQLGFNVNLDV	Mycosubtilin synthase subunit C	0.72	1.5411	0.10329
LTEVIESYLNAHKY	Mycosubtilin synthase subunit C	0.66	0.8240	0.05747

Table S4 Conformational B cell epitopes in the vaccine predicted by ElliPro Server

Sr.No	Residues	No. of residues	Score	3D structure
	_:A1, _:P2, _:P3, _:H4, _:A5, _:L6, _:S7, _:E8, _:A9, _:A10, _:A11, _:K12, _:I13, _:P14, _:D15, _:H16, _:L17, _:E18, _:G19, _:D20, _:I21, _:A22, _:A23, _:Y24, _:F25, _:S26, _:P27, _:R28, _:R29, _:R31	30	0.904	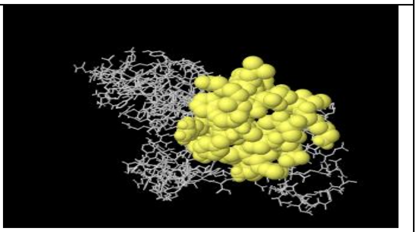
	_:H152, _:V153, _:K154, _:K155, _:I157, _:P158, _:D159, _:V161, _:E162, _:D163, _:D164, _:L165, _:D166, _:G167, _:N168, _:G169, _:K170, _:K171, _:D172, _:C173, _:D174, _:D175, _:D176, _:D177, _:D178, _:D179, _:D180, _:D181, _:G182, _:D185, _:K186	31	0.732	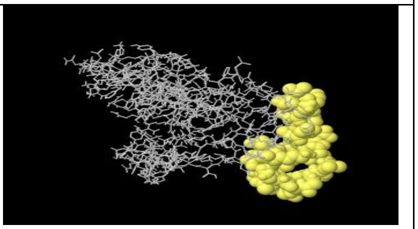
	_:K202, _:K203, _:T205, _:E206, _:V207, _:I208, _:E209, _:S210, _:Y211, _:L212, _:N213, _:A214, _:H215, _:K216, _:Y217	15	0.704	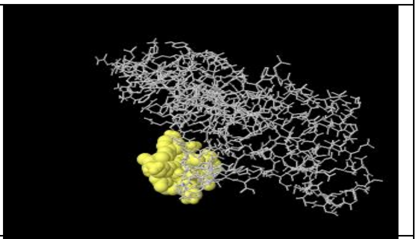
	_:K68, _:A70, _:A71, _:Y72, _:R73, _:Q74, _:L75, _:G76, _:V79, _:A82, _:A83, _:Y85	12	0.644	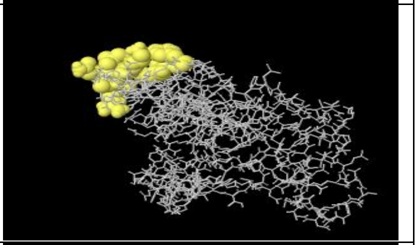
	_:Y30, _:K32, _:L33, _:A34, _:A35, _:Y36, _:E39	7	0.641	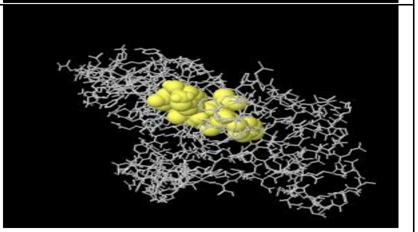
	_:Y60, _:R63, _:E64, _:R67	4	0.593	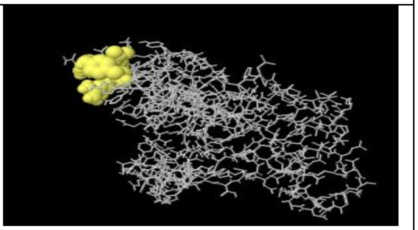

	_:L106, _:S112, _:K113, _:G114, _:P115, _:G116	6	0.508	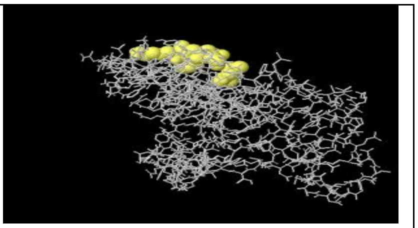

Table S5 Linear B cell epitopes predicted in vaccine by ABCPred server

Sr.No	Peptide	Position	Score	Antigenicity
1.	VGVLISLPAKHVKK	142	0.83	0.7008
2.	DDLDGNGKKDCDDD	163	0.81	1.4896
3.	KKSKGPGPGRNFKL	110	0.81	0.5205
4.	AKIPDHLEGDIAAY	11	0.80	0.9291
5.	PRRYRKLAAYEVEA	27	0.79	0.5366
6.	NLAAYYENPYEHTF	80	0.77	0.7856
7.	YRSRERARKVAAYR	60	0.77	1.0773
8.	IPDYVEDDLDGNGK	157	0.76	1.5153
9.	LGFNVNLAAYYENP	75	0.74	1.1876
10.	PGRNFKLIRSRERA	117	0.73	0.5278
11.	EGDIAAYFSPRRYR	18	0.69	1.1236
12.	EAYAAYFMAVFSHY	43	0.64	0.9670
13.	DDDDDDDGILDKKD	175	0.60	0.9661
14.	LRLSKNKKSKGPGP	104	0.60	1.3415
15.	SHYIAAYRSRERAR	54	0.57	0.8101
16.	KKDCDDDDDDDDGI	170	0.56	1.2584
